# Supplementary material for: Dynamic transcriptomic profiles of zebrafish gills in response to zinc supplementation
Source: BMC Genomics. 2010 Oct 11;11:553. doi: 10.1186/1471-2164-11-553 (PMC3091702; doi:10.1186/1471-2164-11-553)
Supplement: Additional file 2 — Interactive Direct Interaction Network representing the molecular interactions between zinc, copper, iron, calcium and proteins encoded by transcripts changed by zinc supplementation. Mini web-site containing index.html and hyperlinked pages in subdirectory describing a Direct Interaction Network automatically generated based on curated interactions contained within the proprietary PathwayArchitect database. Ovals represent proteins and the circles symbolize metal ions. Objects are coloured by their abundance in zebrafish at the time-point they were significantly different from the control is a scale from -4 fold (dark green) to +4 fold (dark red). Where significant differences were found at more than one time-point, the colour overlay shows expression at the first instance. Dark blue squares denote 'binding', and light blue squares 'expression'; green squares stand for 'regulation', green diamonds for 'metabolism', and green circles for 'promoter binding'. Arrow heads indicate directionality of the interaction where annotated. All nodes and edges can be further interrogated by selecting the relative area of the image. [file 1471-2164-11-553-S2.zip › PathwayArchitect Zn xs DIN/869153.html]

# BINDING:

|  |  |
| --- | --- |
| Type | BINDING |
| Effect | None |


---

|  |  |
| --- | --- |
| Score | 0 |


---

|  |  |
| --- | --- |
| Reference Count | 3 |


---

|  |  |
| --- | --- |
| Mechanism | Unknown |


---

|  |  |
| --- | --- |
| Reference:0 || Sentence | "For protection, especially in conditions of trauma, inflammation and hemolysis, and to maintain iron homeostasis, a high-affinity binding protein, hemopexin, is required." |
| PMID | 10504726 |
| Year | 1999 |
| Journal | Nat Struct Biol |
| RefScore | 0 |
| Source | PArchNLP |
  |
|  |  |


---

|  |  |
| --- | --- |
 Reference:1 || Sentence | "Problems of ferrokinetics, participation of metalloproteins transferrin, ferritin and lactoferrin in metabolism of iron at the step of the metal absorption, transport of iron by means of transferrin, haptoglobin and hemopexin, interaction of transferrin with reticulocytes, deposition of iron in ferritin, mobilization of iron from ferritin via ceruloplasmin are considered." |
| PMID | 3047970 |
| Year | 1988 |
| Species | Human |
| Journal | Vopr Med Khim |
| RefScore | 1 |
| Source | PArchNLP |
  ||  |  |


---

|  |  |
| --- | --- |
 Reference:2 || Sentence | "Cell Physiol., 156:280-285), and the extracellular antioxidant, apo-hemopexin, which binds free heme (iron-protoporphyrin IX)." |
| PMID | 8647924 |
| Year | 1996 |
| Species | Human |
| Journal | J Cell Physiol |
| RefScore | 1 |
| Source | PArchNLP |
  |


---

|  |  |
| --- | --- |
